# Susceptibility of *Phytomonas serpens* to calpain inhibitors in vitro: interference on the proliferation, ultrastructure, cysteine peptidase expression and interaction with the invertebrate host

**DOI:** 10.1590/0074-02760160270

**Published:** 2016-12-01

**Authors:** Simone Santiago Carvalho de Oliveira, Diego de Souza Gonçalves, Aline dos Santos Garcia-Gomes, Inês Correa Gonçalves, Sergio Henrique Seabra, Rubem Figueiredo Menna-Barreto, Angela Hampshire de Carvalho Santos Lopes, Claudia Masini D’Avila-Levy, André Luis Souza dos Santos, Marta Helena Branquinha

**Affiliations:** 1 Universidade Federal do Rio de Janeiro, Instituto de Microbiologia Paulo de Góes, Departamento de Microbiologia Geral, Laboratório de Investigação de Peptidases, Rio de Janeiro, RJ, Brasil; 2 Universidade Federal do Rio de Janeiro, Instituto de Química, Programa de Pós-Graduação em Bioquímica, Rio de Janeiro, RJ, Brasil; 3 Fundação Oswaldo Cruz, Instituto Oswaldo Cruz, Laboratório de Estudos Integrados em Protozoologia, Coleção de Protozoários, Rio de Janeiro, RJ, Brasil; 4 Instituto Federal de Educação, Ciência e Tecnologia, Laboratório de Microbiologia, Rio de Janeiro, RJ, Brasil; 5 Universidade Federal do Rio de Janeiro, Instituto de Microbiologia Paulo de Góes, Departamento de Microbiologia Geral, Laboratório de Bioquímica de Microrganismos, Rio de Janeiro, RJ, Brasil; 6 Centro Universitário Estadual da Zona Oeste, Laboratório de Tecnologia em Cultura de Células, Rio de Janeiro, RJ, Brasil; 7 Fundação Oswaldo Cruz, Instituto Oswaldo Cruz, Laboratório de Biologia Celular, Rio de Janeiro, RJ, Brasil

**Keywords:** Phytomonas, calpain-like proteins, cysteine peptidase, cruzipain, Gp63, Oncopeltus fasciatus

## Abstract

A pleiotropic response to the calpain inhibitor MDL28170 was detected in the tomato parasite *Phytomonas serpens*. Ultrastructural studies revealed that MDL28170 caused mitochondrial swelling, shortening of flagellum and disruption of trans Golgi network. This effect was correlated to the inhibition in processing of cruzipain-like molecules, which presented an increase in expression paralleled by decreased proteolytic activity. Concomitantly, a calcium-dependent cysteine peptidase was detected in the parasite extract, the activity of which was repressed by pre-incubation of parasites with MDL28170. Flow cytometry and Western blotting analyses revealed the differential expression of calpain-like proteins (CALPs) in response to the pre-incubation of parasites with the MDL28170, and confocal fluorescence microscopy confirmed their surface location. The interaction of promastigotes with explanted salivary glands of the insect *Oncopeltus fasciatus* was reduced when parasites were pre-treated with MDL28170, which was correlated to reduced levels of surface cruzipain-like and gp63-like molecules. Treatment of parasites with anti-*Drosophila melanogaster* (Dm) calpain antibody also decreased the adhesion process. Additionally, parasites recovered from the interaction process presented higher levels of surface cruzipain-like and gp63-like molecules, with similar levels of CALPs cross-reactive to anti-Dm-calpain antibody. The results confirm the importance of exploring the use of calpain inhibitors in studying parasites’ physiology.

The genus *Phytomonas* comprises trypanosomatids found in latex, phloem, fruits and seeds of different plant species with a wide geographical distribution (Camargo 1999, Jaskowska et al. 2015). Some species are etiological agents of diseases that affect economically important plants, including coffee, corn, manioc and palms (Jaskowska et al. 2015). *Phytomonas serpens* was isolated for the first time from the sap of tomatoes, but there is no precise information available about its pathogenicity in the fruit, since promastigotes remain compressed around the point of inoculation; however, a loss in both nutritional quality and in economic value added to the product were well documented (Camargo 1999). Due to the facility of in vitro cultivation of this tomato isolate (Camargo 1999), some characteristics of *Phytomonas* physiological properties and the impact of the parasite on the host have been investigated in detail for this species. The transmission of *P. serpens* into tomatoes by *Phthia picta* (Hemiptera: Coreidade) and *Nezara viridula* (Hemiptera: Pentatomidae) and vice versa has been proved, which is difficult to verify experimentally in other phytomonads (Camargo 1999). The phytophagous insect *Oncopeltus fasciatus* is also able to host *P. serpens*, as determined by experimental infection, allowing its use in distinct approaches concerning the interaction of the phytomonad with the salivary gland of the insect before its transmission (Camargo 1999, Jaskowska et al. 2015).

Another aspect that deserves attention in the many studies employing *P. serpens* is the humoral and cellular cross-immunity of this parasite against *Trypanosoma cruzi* and *Leishmania* spp., the causative agents of Chagas’ disease and leishmaniases in humans, respectively, which suggests similarities among their structural components (Breganó et al. 2003, Pinge-Filho et al. 2005, Santos et al. 2007, de Souza et al. 2010). Our group has previously shown that *P. serpens* synthesises metallo- and cysteine-peptidases that are related to leishmanial gp63 and *T. cruzi* cruzipain, respectively, both peptidases displaying virulence-related functions in these pathogenic species (Santos et al. 2007).

Many experimental evidences have demonstrated the important roles that calpain-like proteins (CALPs) may play in trypanosomatids, such as the stage-specific expression in distinct parasites and the differential expression of CALPs in drug-resistant strains (Branquinha et al. 2013). Calpains are neutral, calcium-dependent cysteine peptidases that form one of the most important proteolytic systems of mammalian cells (Goll et al. 2003, Ono & Sorimachi 2012). Numerous functions related to signal transduction, cell motility, differentiation, proliferation, gene expression and apoptosis have been postulated for calpains in the human body (Goll et al. 2003, Ono & Sorimachi 2012). The large and diverse family of CALPs detected in trypanosomatids (Ersfeld et al. 2005) was categorised into five groups, based on their structural features, but the absence of amino acid residues essential for catalytic activity and the moderate overall degree of sequence identity with human calpains suggest that most of these CALPs do not have proteolytic activity (Ersfeld et al. 2005, Branquinha et al. 2013). Non-proteolytic CALPs are likely to function as structural elements and in regulatory processes, and as such a universal function of calpains and CALPs appears to be that of a scaffold by interacting with various molecules, as shown by their wide range of substrate specificity (Tonami et al. 2007).

Some studies from our group using immunoblotting analysis showed that the anti-Dm-calpain antibody, specific against *Drosophila melanogaster* calpain (Emori & Saigo 1994), strongly recognised a polypeptide of approximately 80 kDa in the spent culture medium of the insect trypanosomatid *Angomonas deanei* (formely *Crithidia deanei*), in *Leishmania amazonensis* promastigotes, in *Herpetomonas samuelpessoai* promastigotes and paramastigotes as well as in *T. cruzi* epimastigotes (Branquinha et al. 2013). The calpain inhibitor MDL28170, which is a potent and cell-permeable inhibitor, was able to arrest the growth of *L. amazonensis* and *T. cruzi* in a dose-dependent manner (Branquinha et al. 2013). In addition, we also reported that MDL28170 was able to interfere in many aspects of *T. cruzi* life cycle, which includes the reduction of the viability of infective trypomastigote forms and their interaction with macrophages, besides the inhibition of epimastigotes adhesion to the insect midgut and the differentiation process into metacyclic trypomastigotes (Branquinha et al. 2013). These data point to the importance of the studies concerning the effects of calpain inhibitors in different stages of the parasites’ metabolism.

In the present study, we expanded these findings initially investigating the effects of distinct calpain inhibitors on *P. serpens* growth rate. In addition, the influence of MDL28170 on the ultrastructure of the parasite and on the detection of distinct cysteine peptidase activities was evaluated. We also report the effects of MDL28170 on the expression of CALPs, gp63-like and cruzipain-like proteins in *P. serpens* and the role of these molecules on the interaction with *O. fasciatus* salivary glands.

## MATERIALS AND METHODS

*Parasite and cultivation* - *Phytomonas serpens* (isolate 9T), isolated from tomato (*Lycopersicon esculentum*), is deposited under the accession number COLPROT 189 at Protozoa Collection, Instituto Oswaldo Cruz - Fundação Oswaldo Cruz, Rio de Janeiro, Brazil. The plant flagellate was grown in Warren medium (3.7% brain heart infusion medium supplemented with folic acid 10 µg/L and hemin 1 mg/L) containing 5% (v/v) heat-inactivated fetal bovine serum at 28ºC for two days.

*Measurement of cell growth and light microscopy* - The action of three cell-permeable calpain inhibitors was evaluated upon the growth rate of *P. serpens* promastigote forms: MDL28170 (a reversible peptidomimetic inhibitor, also known as calpain inhibitor III; Z-Val-Phe-CHO; Z = *N*-benzyloxycarbonyl); calpain inhibitor V (an irreversible peptidomimetic inhibitor; Mu-Val-HPh-FMK; Mu = morpholinoureidyl; HPh = homophenylalanyl; FMK = fluoromethylketone); and PD150606 (3-(4-Iodophenyl)-2-mercapto-(Z)-2-propenoic acid, a non-competitive calpain inhibitor directed towards the calcium-binding sites of calpain). These compounds (Calbiochem, San Diego, CA, USA) were dissolved in dimethylsulfoxide (DMSO; Sigma-Aldrich, Saint Louis, MO, USA) at 5 mM.

Briefly, promastigotes were counted using a Neubauer chamber and resuspended in fresh medium to a final concentration of 10^6^ viable promastigotes per milliliter. The viability was assessed by mobility and lack of staining after challenging with Trypan blue (D’Avila-Levy et al. 2006). Each calpain inhibitor was added to the culture at final concentrations in the 10-70 mM range. A dilution of DMSO corresponding to that used to prepare the highest drug concentration was assessed in parallel. After 24, 48, 72 and 96 h of incubation at 28ºC, the number of viable, motile promastigotes was quantified. Alternatively, parasites grown for 72 h in the absence and in the presence of each calpain inhibitor were washed five times in cold phosphate-buffered saline (PBS; 150 mM NaCl, 20 mM phosphate buffer, pH 7.2) prior to resuspension in a drug-free fresh medium and allowed to grow for another 72 h, in order to evaluate the cytocidal or cytostatic effect. The number of live promastigotes was evaluated under optical microscopy at 24 h intervals and the 50% inhibitory concentration (IC_50_) was evaluated after 48 h (mid-log phase growth). This value was determined by linear regression analysis, by plotting the log number of viable promastigotes versus drug concentration by use of Origin Pro 7.5 computer software.

Light microscopy evaluation was performed in order to detect some possible alterations on parasite morphology after treatment with MDL28170 at ½ x IC_50_, IC_50_ or 2 x IC_50_ values for 48 h. In this context, the parasites were stained with Giemsa and then observed under a Zeiss microscope (Axioplan, Oberkochen, Germany).

*Scanning and transmission electron microcopy* - Promastigote forms of *P. serpens* (10^6^ cells/mL) were cultured in Warren medium for 48 h supplemented or not with the calpain inhibitor MDL28170 at the IC_50_ value. For the observation of the ultrastructure modifications by scanning electron microscopy, promastigotes were fixed for 40 min at 25ºC with 2.5% glutaraldehyde in 0.1 M cacodylate buffer, pH 7.2. After fixation, cells were washed in cacodylate buffer and post-fixed with a solution of 1% OsO_4_, 0.8% potassium ferrocyanide and 5 mM CaCl_2_ in the same buffer 20 min at 25ºC. Cells were dehydrated in graded series of acetone (30-100%) and then dried by the critical point method, mounted on stubs, coated with gold (20-30 nm) and observed in a Jeol JSM 6490LV scanning electron microscope (Massachusets, USA) (Portes et al. 2012). For transmission electron microscopy observation, cells over coverslips were fixed and post-fixed after 48 h of drug treatment as described above, dehydrated in an ascending acetone series and embedded in PolyBed 812 resin. Ultrathin sections were stained with uranyl acetate and lead citrate and examined in Jeol JEM1011 transmission electron microscope (Tokyo, Japan) at Plataforma de Microscopia Eletrônica, IOC, FIOCRUZ (Salomão et al. 2013).

*Flow cytometry analysis* - Promastigotes of *P. serpens* (10^6^ cells) were incubated or not with MDL28170 at the IC_50_ value for 24 h at 28ºC. Viability of cells was confirmed by mobility and lack of staining after challenging with Trypan blue. Thereafter, cells were fixed for 15 min in 0.4% paraformaldehyde in PBS (pH 7.2) at room temperature, followed by extensive washing in the same buffer. Alternatively, fixed cells were permeabilised by 0.01% Triton X-100 in PBS for 15 min at room temperature and then washed twice in PBS. The fixed and permeabilised cells maintained their morphological integrity, as verified by optical microscopic observation. Cells were then incubated for 1 h at room temperature with a 1:250 dilution of the following rabbit antibodies: anti-Dm-calpain (polyclonal, raised against the 70-kDa C-terminal region of calpain from *Drosophila melanogaster* and kindly donated by Dr Yasufumi Emori - Department of Biophysics and Biochemistry, Faculty of Sciences, University of Tokyo, Japan) (Emori & Saigo 1994); anti-CAP5.5 (monoclonal, raised against the cytoskeleton-associated protein from *T. brucei* and kindly provided by Dr. Keith Gull - Sir William Dunn School of Pathology, University of Oxford, England) (Hertz-Fowler et al. 2001); anti-CDPIIb (polyclonal, raised against *Homarus americanus* calpains and kindly donated by Dr Donald L Mykles - Colorado State University, USA); anti-gp63 (raised against the recombinant gp63 molecule from *L. mexicana* and kindly provided by Dr Peter Overath - Max-Planck-Institut für Biologie, Abteilung Membranbiochemie, Germany) and anti-cruzipain (raised against cruzipain from *T. cruzi* and kindly provided by Dr Ana Paula Lima - Instituto Biofísica Carlos Chagas Filho, Universidade Federal do Rio de Janeiro, Brazil). Cells were then incubated for an additional hour with a 1:100 dilution of fluorescein isothiocyanate (FITC)-labeled goat anti-rabbit immunoglobulin G (IgG) (Sigma-Aldrich). Finally, cells were washed three times in PBS and analysed in a flow cytometry (FACSCalibur, BD Bioscience, USA) equipped with a 15 mW argon laser emitting at 488 nm. Non-treated cells and those treated with the secondary antibody alone were run in parallel as controls (D’Avila-Levy et al. 2006).

*Western blotting* - *P. serpens* promastigote forms were subjected or not to MDL28170 treatment at the IC_50_ concentration for 24 h. Cells (2 × 10^8^) were then collected by centrifugation at 3000 x g for 5 min at 4ºC, washed three times with cold PBS and lysed with 100 µL of sodium dodecyl sulfate-polyacrylamide gel electrophoresis (SDS-PAGE) sample buffer (62 mM Tris-HCl, pH 6.8, 2% SDS, 25% glycerol, 0.01% bromophenol blue and 1 mM β-mercaptoethanol). Immunoblot analysis was performed with total cellular extracts (equivalent to 5 × 10^6^ cells) as previously described (D’Avila-Levy et al. 2006). Proteins were separated in 10% SDS-PAGE under reducing conditions and the polypeptides were electrophoretically transferred at 4ºC at 100 V/300 mA for 2 h to nitrocellulose membranes. The membrane was blocked in 10% low-fat dried milk dissolved in Tris-buffered saline (TBS; 150 mM NaCl; 10 mM Tris, pH 7.4) containing 0.05% Tween 20 (TBS/Tween) for 1 h at room temperature. Membranes were washed three times (10 min each) with the blocking solution and incubated for 2 h with the primary antibodies anti-Dm-calpain, anti-CAP5.5 and anti-CDPIIb at 1:1000 dilution. The secondary antibody used was peroxidase-conjugated goat anti-rabbit IgG at 1:25,000 followed by chemiluminescence immunodetection after reaction with ECL reagents. An anti-alpha-tubulin monoclonal antibody (Sigma-Aldrich) at 1:1000 dilution was also used as a control for sample loading in the immunoblot. The relative molecular mass of the reactive polypeptides was calculated by comparison with the mobility of SDS-PAGE standards and the densitometric analysis was performed using the ImageJ program.

Alternatively, *P. serpens* promastigote forms were subjected or not to MDL28170 treatment at the ½ x IC_50_, IC_50_ or 2 x IC_50_ values of MDL28170 for 4 h. Cells (2 × 10^8^) were collected and processed as described above and subjected to immunoblot analysis using anti-cruzipain as the primary antibody.

*Sequence data analysis* - A search for Dm-calpain, CAP5.5 and CDPIIb homologous proteins in the streamlined genome of *Phytomonas* sp. isolate EM1 was conducted using the BlastP algorithm and the *nr* database at National Center for Biotechnology Information (NCBI, GenBank). The following queries were compared in a BlastP analysis against *Phytomonas* sp. isolate EM1 proteins found in GenBank database: the fragment of the AHN56408.1 protein used to generate the Dm-calpain antibody (Emori & Saigo 1994), AAG48626.1 protein for *T. brucei* anti-CAP5.5 calpain and AAM88579.1 for anti-CDPIIb lobster calpain. The theoretical molecular masses of homologous proteins were calculated using the ExPASy Server facilities (http://expasy.org). Identification of conserved domains was performed using the CDD tool at NCBI (Marchler-Bauer et al. 2015). Moreover, an additional search for the total number of cysteine peptidases from *Phytomonas* sp. isolate EM1 was conducted using “cysteine-type endopeptidase activity” as query on the UniProt Knowledgebase (UniProtKB). Blast analysis for all queries was performed on UniProtKB to search similarities with *T. cruzi* cruzipain.

*Confocal fluorescence microscopy* - In this set of experiments, promastigotes of *P. serpens* (10^6^ cells) pre-treated or not with the calpain inhibitor at the IC_50_ value were fixed, permeabilised and processed as described for the flow cytometry analysis using a 1:250 dilution of the anti-cruzipain antibody. For anti-Dm-calpain antibody, cells were not permeabilised. Subsequently, cells were incubated with the FITC-labeled anti-IgG secondary antibody as well as with 4’,6-diamidino-2-phenylindole (DAPI) at 10 µg/µL for 15 min to stain nucleus and kinetoplast. Cells were then washed three times in PBS and observed in a Leica TCS SP5 confocal microscope.

*Cysteine peptidase assays* - Calcium-dependent cysteine peptidase activity over two fluorogenic substrates was determined using *P. serpens* promastigotes that were cultured in the absence (control) or in the presence of the ½ x IC_50_, IC_50_ or 2 x IC_50_ values of MDL28170 for 4 h. Whole parasite cellular extracts were obtained by repeated freeze-thawing cycles of 10^7^ viable cells in a buffer containing 70 mM imidazole, 2 mM dithiotreitol (DTT), 1% CHAPS (3-[(3-cholamidopropyl)dimethylammonio]-1-propanesulfonate), pH 7.0. Then, the cellular extract was centrifuged at 10,000 x g for 30 min at 4ºC, and the supernatant immediately used to determine the protein content and the proteolytic activity. The protein concentration was determined by the method described by Lowry and co-workers (Lowry et al. 1951), using bovine serum albumin (BSA) as standard. The cleavage of the fluorogenic substrates Z-Leu-Tyr-AMC (AMC = amidomethylcoumarin) and Z-Leu-Leu-Val-Tyr-AMC (Sigma-Aldrich), commonly used to measure calpain activity (Atsma et al. 1995), was monitored continuously in a spectrofluorometer (SpectraMax Gemini XPS, Molecular Devices, CA, USA) using an excitation wavelength of 380 nm and an emission wavelength of 460 nm. A 5-mM stock solution of each fluorogenic substrate was prepared in DMSO. The reaction was started by the addition of each substrate (20 µM) to the parasite extract (10 μg protein) in a total volume of 60 µL of a buffer containing 70 mM imidazol, 2 mM DTT, 1% CHAPS, pH 7.0, in the presence or in the absence of 100 mM calcium chloride, the selective and irreversible cysteine peptidase inhibitor *trans*-epoxysuccinyl L-leucylamido-(4-guanidino) butane (E-64) at 10 µM, and the calcium chelator ethylene glycol-bis(β-aminoethyl ether)-N,N,N′,N′-tetraacetic acid (EGTA) at 1 mM. The reaction mixture was incubated at 37ºC for 1 h. The assays were controlled for self-liberation of the fluorophore over the same time interval.

In parallel, the hydrolysis of the fluorogenic substrate Z-Phe-Arg-AMC (Sigma-Aldrich), commonly used to detect cathepsin B and L acitivities, including cathepsin L-like cruzipain (Cazzulo et al. 1990), was measured using *P. serpens* promastigotes that were cultured in the same conditions, *i.e.*, in the absence (control) or in the presence of the ½ x IC_50_, IC_50_ or 2 x IC_50_ values of MDL28170 for 4 h. Whole parasite cellular extracts were obtained as described above. The reaction was started by the addition of the fluorogenic substrate (20 µM, starting from a 5-mM stock solution in DMSO) to the parasite extract (10 μg protein) in a total volume of 60 µL of 50 mM sodium phosphate buffer, pH 5.0, containing 2 mM DTT, in the absence or in the presence of 10 µM E-64. The reaction mixture was incubated at 37ºC for 1 h. The assays were also controlled for self-liberation of the fluorophore over the same time interval.

Cysteine peptidase activities were also assayed in gelatin-containing SDS-PAGE (Heussen & Dowdle 1980). After incubation of *P. serpens* promastigotes with the ½ x IC_50_, IC_50_ or 2 x IC_50_ values of MDL28170 for 4 h, cells were washed three times in PBS and then lysed by the addition of 0.1% SDS. Cells were broken in a vortex by alternating 1-min shaking and 2-min cooling intervals, followed by centrifugation at 10,000 x g for 30 min at 4ºC, in order to obtain the whole parasite cellular extracts. Samples containing 50 µg of protein of each system were resuspended in SDS-PAGE sample buffer (125 mM Tris, pH 6.8, 4% SDS, 20% glycerol and 0.002% bromophenol blue). Peptidases were assayed and characterised by 10% SDS-PAGE with 0.1% co-polymerised gelatin as substrate. After electrophoresis at a constant voltage of 120 V at 4ºC, SDS was removed by incubation with 10 volumes of 2.5% Triton X-100 for 1 h at room temperature under constant agitation. Then, the gels were incubated at 37ºC in 50 mM sodium phosphate buffer supplemented with 2 mM DTT, pH 5.0, for 48 h, to promote the proteolysis. The gels were stained for 2 h with 0.2% Coomassie brilliant blue R-250 in methanol-acetic acid-water (50:10:40) and destained overnight in a solution containing methanol-acetic acid- water (5:10:85), to intensify the digestion halos. The molecular masses of the peptidases were estimated by comparison with the mobility of low molecular mass standards.

*Interaction with Oncopeltus fasciatus salivary glands* - A milkweed bug (*O. fasciatus*) culture kit was purchased from Carolina Biological Supply Company, Burlington, North Carolina, USA. These insects originated the colony maintained at Laboratório de Bioquímica de Microrganismos (Instituto de Microbiologia Paulo de Góes, UFRJ, Brazil). Adult *O. fasciatus* insects (Hemiptera: Lygaeidae) were kept in plastic pitchers under a 12 h light/dark cycle at 28^o^C with 70-80% relative humidity and fed with peeled and toasted sunflower seeds and distilled water (Romeiro et al. 2000).

*P. serpens* (10^7^ cells) was cultured in Warren medium in the absence (control) or the presence of ½ x IC_50_, IC_50_ or 2 x IC_50_ values of MDL28170 for 4 h. A dilution of DMSO corresponding to that used to prepare the highest drug concentration was assessed in parallel. Alternatively, parasites were cultured in the presence of the IC_50_ value of MDL28170 for 24 h. The viability of the organisms throughout the experiment was assessed by mobility and Trypan blue dye exclusion (D’Avila-Levy et al. 2006). MDL28170-treated and control cells were then washed three times in cold PBS, resuspended in PBS (5.0 x 10^6^ cells in 100 µL) and added to dissected salivary glands (five per group). The parasites were allowed to bind for 1 h at room temperature in PBS. After the interaction period, the salivary glands were extensively washed with PBS, individually transferred to microcentrifuge tubes containing 50 µL of PBS and homogenised (D’Avila-Levy et al. 2006). The released trypanosomatids were counted in a Neubauer chamber. The results are shown as the mean ± standard error of the mean of three experiments.

In order to evaluate the capacity of anti-Dm-calpain antibody to interfere in the interaction of promastigotes with the salivary glands, an agglutination assay was performed by incubating 100 µL of a suspension containing 10^8^ parasites/mL with different dilutions (1:125, 1:250, 1:500, 1:1000) of anti-Dm-calpain antibody. The agglutination was evaluated after 1 h at room temperature by comparison with untreated parasites observed in a Zeiss Axiovert light inverted microscope. After this step, 10^7^ cells were treated with anti-Dm-calpain antibody at dilutions that did not promote agglutination (1:250, 1:500 and 1:1000 dilutions) or with a rabbit non-immune IgG antibody (1:250 dilution) for 1 h at room temperature. Cells were then allowed to interact with *O. fasciatus* salivary glands as described in the previous paragraph.

In order to analyse the possible modulation on the expression of surface gp63-like, cruzipain-like and CALPs cross-reactive to anti-Dm-calpain antibody in *P. serpens* after in vitro interaction with *O. fasciatus* salivary glands, the parasite cells released after the interaction step were washed in PBS, fixed and processed for flow cytometry, as described above, using anti-gp63, anti-cruzipain and anti-Dm-calpain antibodies.

*Statistical analysis* - All experiments were performed in triplicate, in three independent experimental sets. The data were analysed statistically by means of Student’s *t* test using EPI-INFO 6.04 (Database and Statistics Program for Public Health) computer software. *P* values of 0.05 or less were considered statistically significant.

## RESULTS AND DISCUSSION

*Effects of calpain inhibitors on the growth rate of P. serpens* - The first step of this study was to evaluate the efficacy of calpain inhibitors in arresting *P. serpens* growth. The reversible and competitive calpain inhibitor MDL28170 at 30, 50 and 70 µM promoted reduced levels of growth that were statistically significant after 48 h (Supplementary data, Fig. 1). The IC_50_ value determined after 48 h of cultivation was 30.9 µM. Comparing the results for MDL28170 in the present paper with those published previously by our group, the parasite presented susceptibility similar to that of *T. cruzi* (IC_50_ value 31.7 µM), while for *L. amazonensis* the activity of MDL28170 was much more pronounced (IC_50_ value 19 µM) (Branquinha et al. 2013).

Although MDL28170 is a prototypical calpain inhibitor (Branquinha et al. 2013), its possible action against cysteine peptidases other than calpains, such as cathepsin B, cannot be completely ruled out due to the similarity of the active site among different classes of cysteine peptidases (Donkor 2015). Several calpain inhibitors were reported in the last years, and their potential therapeutic use is explored for the treatment of several human pathophysiological events in which calpains have been implicated (Donkor 2015). To further substantiate the inhibitory effect of MDL28170 on *P. serpens* promastigotes growth, the action of two additional calpain inhibitors was investigated against this parasite: the non-competitive calpain inhibitor PD150606 and the irreversible calpain inhibitor V.

In this comparative analysis, MDL28170 inhibited *P. serpens* growth at rates that are not significantly different from calpain inhibitor V (IC_50_ value 32.3 µM) but different from that found to PD150606 (IC_50_ value 42.8 µM) (Supplementary data, Fig. 1). Differences in the degree of inhibition of calpain activity might be explained by differences in the chemical structure, mechanisms of action or specificity of calpain inhibitors for a particular calpain structure (Donkor 2015). This is an important issue, especially for invertebrates and lower eukaryotes displaying non-typical calpains, many of them probably with no proteolytic activity (Ersfeld et al. 2005). However, even not displaying proteolytic activity, the detection of their expression may point to organism-specific functions for these proteins (Goll et al. 2003), and it has been speculated that calpains devoid of enzymatic activity are involved in regulatory processes and as structural elements (Ersfeld et al. 2005, Tonami et al. 2007). In this sense, the similarity in IC_50_ values for the active site-directed calpain inhibitors MDL28170 and calpain inhibitor V is suggestive of the action of both compounds against similar targets in the parasite. On the other hand, PD150606 selectively inhibits mammalian calpains relative to other peptidases, such as cathepsin B and cathepsin L, since it targets the calcium-binding domains in both calpain subunits that are essential for enzymatic activity and not found in cathepsins (Donkor 2015). However, these calcium-binding motifs are absent in trypanosomatid CALPs, although amino acid residues that are critical for binding of calcium in mammalian calpains are partially conserved in some kinetoplastid sequences (Ersfeld et al. 2005). In addition, it was recently proven that PD150606 must be also acting at a site on the peptidase core domain of calpains to perform its inhibition (Low et al. 2014). These aspects could explain the differences between the inhibitory effects of MDL28170 and calpain inhibitor V on *P. serpens* growth in comparison to PD150606. The answer to this question demands further experiments in order to exclude off-target effects of calpain inhibitors.

DMSO, the solvent used to dissolve the calpain inhibitors, had no effect on the parasite multiplication when added in the volume corresponding to the highest concentration of each inhibitor (data not shown). In addition, the anti-phytomonad activity of the calpain inhibitors was cytostatic, since *P. serpens* cells pre-treated for 72 h with each calpain inhibitor at 70 µM resumed growth when subcultured in a drug-free fresh medium (data not shown). The comparison between the growth rates of *P. serpens* control cells and those cells that resumed growth after the 72 h contact with MDL28170 at 70 µM demonstrated that the latter had a much lower growth rate in drug-free fresh medium, with reduced values varying from 52.9% after 24 h of incubation to 71.7% after 96 h of incubation, when compared to control cells (data not shown).

Based on the strongest effect of MDL28170 in diminishing the growth of *P. serpens* in comparison to calpain inhibitor V and PD150606, we decided to investigate the effects of MDL28170 on the ultrastructure as well as on different aspects of the parasite’s physiology.

*Effects of MDL28170 on P. serpens morphology and ultrastructure* - Light microscopy analysis of *P. serpens* cells treated with MDL28170 at the IC_50_ and 2 × IC_50_ values revealed some morphological alterations in comparison to the typical promastigote appearance: parasites became round in shape, with reduced cell size and swollen of the cellular body, as well as shortening and loss of the flagellum (Supplementary data, Fig. 1). These morphological alterations were not observed after treatment of parasite cells with the ½ x IC_50_ value of the calpain inhibitor (Supplementary data, Fig. 1).

**Fig. 1:**
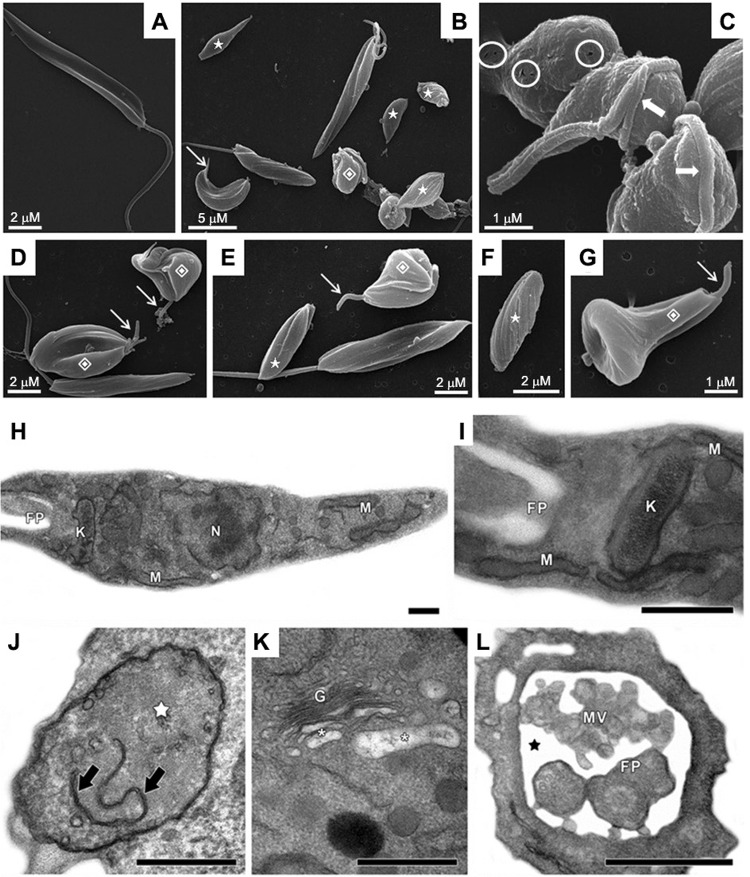
: scanning electron microscopy (SEM, A-G) and transmission electron microscopy (TEM, H-L) of *Phytomonas serpens* after treatment with MDL28170. In untreated, control promastigotes (A), note the elongated body and emerging flagellum in SEM. Treatment with the IC50 value of MDL28170 for 48 h (B-G) caused rounding of the parasite body (B, C), shortening (thin arrows) or complete loss (stars) of the flagellum, cell surface discontinuity (circles) and appearance of cells with bizarre shape (diamonds) and with flagellum contorting the cell body (thick arrows). Control parasites in TEM (H, I) showed typical morphology of nucleus (N), mitochondrion (M), kinetoplast (K) and flagellar pocket (FP). The treatment of promastigotes with the IC50 value of MDL28170 for 48 h (J-L) led to the mitochondrial swelling (white star) with membranes in the organelle matrix (black arrows), the disruption of *trans*-Golgi (G) with peripheral dilation of cisternae (white asterisks), as well as the dilation of flagellar pocket (black star) presenting microvesicules (MV) inside. Bars in TEM: 0.5 µM.

Ultrastructure of non-treated cells was initially compared by scanning electron microscopy with the ultrastructure of parasites treated for 48 h with the IC_50_ value of MDL28170 (Fig. 1A-G). The results showed that control, non-treated parasites, retained their normal features, like a stable and regular cell surface, the typical elongated promastigote shape and a long flagellum (Fig. 1A). The treatment of *P. serpens* promastigotes with MDL28170 led to several morphological changes, such as rounding of the parasite cell body and cell shrinkage (Fig. 1B-C), possibly due to osmotic stress, as previously observed in *P. serpens* after treatment with cysteine peptidase inhibitors such as cystatin, antipain and iodoacetamide (Santos et al. 2006). Promastigotes exposed to the calpain inhibitor also presented complete loss or shortening of the flagellum (Fig. 1B, D, G), cell surface discontinuity (Fig. 1C) and appearance of cells with bizarre shape and with flagellum contorting the cell body (Fig. 1B-G).

Transmission electron microscopy analysis pointed to ultrastructural changes after 48 h of incubation with the calpain inhibitor at the IC_50_ value (Fig. 1J-L) in comparison to the typical morphology of non-treated parasites (Fig. 1H-I). MDL28170-treated cells showed mitochondrial swelling with membranar structures and scarce cristae in the organelle matrix (Fig. 1J). In addition, the calpain inhibitor also induced *trans* Golgi disruption with peripheral dilation of cisternae (Fig. 1K), as well as the appearance of the dilation of flagellar pocket, where several microvesicules were found (Fig. 1L).

The disruption of *trans* Golgi cisternae in *P. serpens* is consistent with the reported effect of this calpain inhibitor in *T. cruzi* epimastigotes (Branquinha et al. 2013), where it was postulated that Golgi alterations could be correlated to the inhibition of metacyclogenesis, in which trypomastigote-specific proteins must be synthesised. Engel et al. (1998) have previously shown that peptidomimetic cysteine peptidase inhibitors were able to prevent the normal autocatalytic processing and trafficking of cruzipain, the major cysteine peptidase of *T. cruzi*, within the Golgi apparatus, which led to massive accumulation of the precursor protein in the Golgi complex, culminating with peripheral distention of cisternae. In this way, a possible explanation to the alterations in Golgi cisternae previously observed by our group would also be the non-specific action of MDL28170 in the intracellular trafficking of *T. cruzi* cruzipain, and it is possible to speculate that the same happens to the cruzipain-like molecules detected in *P. serpens*. The possible broad spectrum of activity of the calpain inhibitor precluded the identification of the primary target of this compound at the ultrastructural level, and demanded further experiments, which were performed in this work.

**Fig. 2:**
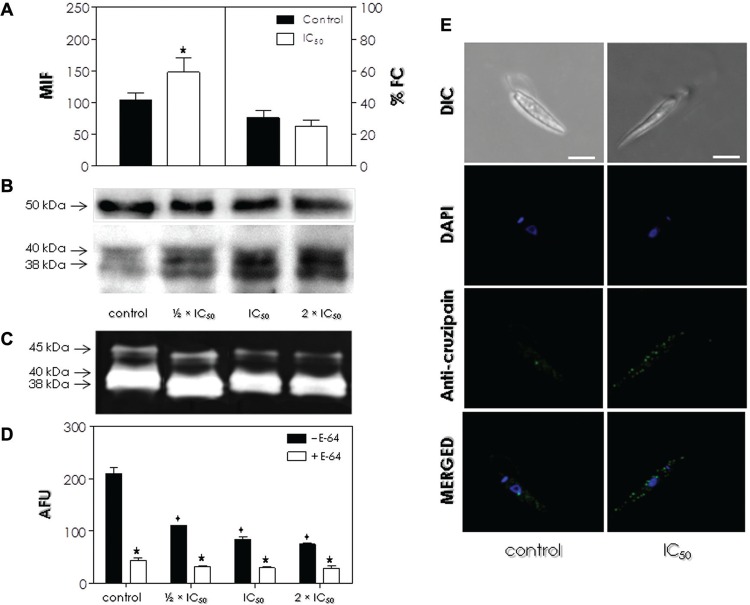
: effect of MDL28170 on the expression of intracellular cruzipain-like molecules and cysteine peptidase activity by *Phytomonas serpens.* (A) Promastigotes were cultured in the absence (control) or the presence of the IC50 value of MDL28170 for 24 h and then fixed, permeabilised cells were processed for flow cytometry analysis using anti-cruzipain antibody. Data are expressed as the percentage of fluorescent cells (% FC) and also the mean of fluorescence intensity (MFI) levels. Representative data of the analysis of 10,000 cells from three experiments are shown. The star highlights significant different value for MFI level between MDL28170-treated and control cells (p < 0.05). (B-D) Promastigotes were cultured in the absence (control) or the presence of the ½ x IC50, IC50 or 2 x IC50 values of MDL28170 for 4 h. Western blotting (B) shows the proteins recognised in the whole cellular extract by the anti-cruzipain antibody (lower panel). Anti-tubulin monoclonal antibody was also used as a control for sample loading in the blots, revealing a protein band of 50 kDa, detected in all strains (upper panel). The peptidase profiles in cell extracts were analysed by means of gelatin-SDS-PAGE (C); gel strips were incubated at 37ºC in phosphate buffer, pH 5.0, supplemented with DTT. Molecular masses of the proteins (B) and peptidases (C), expressed in kDa, are represented on the left. The enzymatic activity in parasite lysates was assessed by measuring the hydrolysis of Z-Phe-Arg-AMC (D) in the absence (- E-64) or the presence of 10 µM E-64 (+ E-64). Results are expressed as arbitrary fluorescence units (AFU) and the values represent the mean ± standard deviation of three independent experiments performed in triplicate. The stars highlight significant different values in the hydrolytic activity in the absence or the presence of E-64, while diamonds denote significant differences between MDL28170-treated and control cells in the absence of E-64 (p < 0.05). (E) Differential interference contrast microscopy (DIC) and confocal scanning images showing the double labeling of *P. serpens* cells for anti-cruzipain antibody (green labeling) and DAPI (blue labeling). The labeling in permeabilised cells suggests the distribution of cruzipain-like proteins throughout the intracellular region, and DAPI stained the nucleus (n) and the kinetoplast (k). At bottom, the overlay of anti-cruzipain antibody and DAPI labeling. Bars: 5 µM.

*Effects of MDL28170 on the expression of cysteine peptidases in P. serpens* - We next decided to investigate the possible role of MDL28170 in processing cruzipain-like peptidases in phytomonads. In the flow cytometry analysis, cells pre-treated with the calpain inhibitor at the IC_50_ value for 24 h presented a significantly increased MFI (mean of fluorescence intensity) level (approximately 30%) of intracellular cruzipain-like proteins in comparison to control cells, but no significant difference in the percentage of fluorescent cells (Fig. 2A). The higher expression of these proteins was confirmed by Western blotting analysis, in which cells that were pre-treated with ½ x IC_50_, IC_50_ and 2 x IC_50_ values of MDL28170 for 4 h presented a progressively increased amount of two proteins at 38 kDa and 40 kDa that cross-reacted with anti-cruzipain antibody (Fig. 2B).

When the cell-associated proteolytic profile of *P. serpens* was checked in gelatin-containing SDS-PAGE under conditions that favor classical cysteine peptidase activity such as cruzipain, as pH 5.0 and in the presence of the reducing agent DTT, two major bands were detected at 38 kDa and 40 kDa as well as a minor doublet band at 45 kDa (Fig. 2C). However, pre-treatment of cells with ½ x IC_50_, IC_50_ and 2 x IC_50_ values of MDL28170 for 4 h induced an apparent reduction in the proteolytic activity of all the bands detected, which could not be quantified by this technique due to its qualitative approach. Since calpains display neutral pH optimum, these bands must not correspond to calpain activity. Subsequently, the quantification of the proteolytic activity against Z-Phe-Arg-AMC, a fluorogenic substrate commonly used to detect cathepsin B and L cysteine peptidase activity, including cruzipain (Cazzulo et al. 1990), but not hydrolysed by calpains (Edelstein et al. 1996), was tested. Pre-treatment of promastigotes with MDL28170 in the same conditions described above promoted a reduction in the hydrolysis of the fluorogenic substrate when compared to non-treated cells, with a clear dose-dependent effect (Fig. 2D). The addition of the cysteine peptidase inhibitor E-64 in the reaction mixture diminished significantly the hydrolytic activity both in control and MDL28170-treated cells, corroborating the predominance of cysteine peptidase activities (Fig. 2D).

The lower proteolytic activity of cruzipain-like molecules, as detected by gelatin-SDS-PAGE and by the hydrolysis of a cathepsin B- and L-specific fluorogenic substrate, implies that pre-treatment of *P. serpens* with the calpain inhibitor induces the expression of higher levels of cruzipain-like molecules devoid of proteolytic activity, and the ultrastructural alterations detected in MDL28170-treated promastigotes may indicate the accumulation of these molecules, in an unprocessed form, within the Golgi apparatus. These results are consistent to that described for *T. cruzi* after treatment with cysteine peptidase inhibitors (Engel et al. 1998). However, cruzipain-like proteins gene expression or protein translation must be employed in order to a proper response to this possibility.

Additionally, cells pre-treated or not with the IC_50_ value of the calpain inhibitor for 24 h and incubated with anti-cruzipain antibody were analysed by confocal microscopy. As visualised in the flow cytometry (Fig. 2A) and Western blotting (Fig. 2B) analyses, immunofluorescence images also confirmed the increase in the expression of cruzipain-like proteins after treatment with the IC_50_ value of MDL28170 in comparison to control cells, with a homogenous distribution throughout the cell body (Fig. 2E).

*Detection of calcium-dependent cysteine peptidase activity in P. serpens extracts and influence of MDL28170 on the proteolytic activity* - Once the presence of cruzipain-like proteins was confirmed in *P. serpens* extracts and their expression and activity was affected by the pre-treatment of parasite cells with the calpain inhibitor (Fig. 2), we decided to check whether the parasite soluble extract was able to hydrolyse two fluorogenic substrates under conditions that favor the proteolytic activity of calcium-dependent cysteine peptidases, *i.e.*, in the presence of calcium ions at pH 7.0. The proteolytic activity present in the whole cellular extract was able to cleave both peptide substrates under these conditions, with best hydrolysis rates for Z-Leu-Tyr-AMC (Supplementary data, Fig. 2). However, the absence of calcium reduced the proteolytic activity by approximately 61% for the Z-Leu-Tyr-AMC substrate and by approximately 48% for the Z-Leu-Leu-Val-Tyr-AMC substrate (Supplementary data, Fig. 2). In addition, the presence of the cysteine peptidase inhibitor E-64 powerfully inhibited the hydrolytic activity on both substrates irrespective of the presence of calcium, suggesting the involvement of cysteine peptidases in this process. Concomitantly, the calcium chelator EGTA promoted a 40% inhibition of the proteolytic activity on both substrates in the absence of calcium. In this case, the highest percentages of inhibition were found when calcium was added to the reaction mixture: a 56% reduction for the Z-Leu-Tyr-AMC substrate and a 70% reduction for the Z-Leu-Leu-Val-Tyr-AMC substrate (Supplementary data, Fig. 2).

When *P. serpens* cells were pre-treated with ½ x IC_50_, IC_50_ and 2 x IC_50_ values of MDL28170 for 4 h and then the soluble extract was prepared, hydrolysis of the Z-Leu-Tyr-AMC substrate in the presence of calcium was lowered by approximately 57.7%, 63.5% and 75.2%, respectively (Supplementary data, Fig. 2). A similar result was found with the Z-Leu-Leu-Val-Tyr-AMC substrate, for which a reduction in the release of AMC of approximately 33.2%, 50.7% and 64.3% was detected, respectively. The results also showed that when calcium was removed from the reaction mixture, the hydrolysis of both substrates was similar to control cells, except for the pre-treatment of cells with 2 x IC_50_ values of MDL28170, for which a significant reduction in the hydrolysis of both substrates was detected (Supplementary data, Fig. 2).

These data encouraged us to postulate that probably two distinct cysteine peptidase activities were detected in these experimental conditions: one that is calcium-dependent and affected by the pre-treatment with the calpain inhibitor, and a residual hydrolytic activity that is calcium-independent, only affected by the previous incubation in higher concentrations of MDL28170. The latter can be probably associated to the 40-kDa and 38-kDa cruzipain-like cysteine peptidases previously detected by our group in *P. serpens* (Santos et al. 2006, 2007) and also detected in the present paper.

*Effects of MDL28170 on the expression of calpain-like molecules in P. serpens* - Flow cytometry and Western blotting analyses were used to study the presence of CALPs in *P. serpens* and to evaluate the effect of MDL28170 on the expression of these CALPs. Flow cytometry analysis of *P. serpens* promastigote cells treated or not with the IC_50_ value of the calpain inhibitor was performed using three anti-calpain antibodies from distinct origins and different specificities. In this analysis, the binding of the three anti-calpain antibodies was significantly enhanced when control, ﬁxed cells were permeabilised, which indicates that CALPs are located mainly in intracellular compartments: more than 70% of cells were labeled with each antibody (Supplementary data, Table I). In non-permeabilised cells, the anti-CAP5.5 and anti-CDPIIB antibodies binding was drastically diminished, but 39% of cells were positively labeled with anti-Dm-calpain antibody. The latter antibody also displayed a significant much higher MFI value (191.2) in the intracellular milieu and on the cell surface (MFI 37.1), in comparison to anti-CAP5.5 (MFI 26.4 and 15.2, respectively) and anti-CDPIIb (MFI 34.4 and 15.5, respectively) antibodies. Anti-calpain antibodies were already found to react with the cell surface of *T. cruzi* epimastigotes and *L. amazonensis* promastigotes, although the antibody binding was stronger intracellularly (Branquinha et al. 2013).

When *P. serpens* cells were treated with MDL28170, there were no significant differences in labeling to control, non-permeabilised cells. However, a significant reduction was detected in MFI levels to anti-Dm-calpain antibody between control cells (MFI 37.1) and MDL28170-treated cells (MFI 29.8) (Supplementary data, Table I). The permeabilization with Triton X-100 led to a significant decrease in MFI levels in MDL28170-treated cells in comparison to control cells: when anti-Dm-calpain antibody was employed, MFI was reduced from 191.2 to 110.9, while for anti-CAP5.5 antibody MFI levels decreased from 26.4 to 17.8 and for anti-CDPIIb antibody the MFI levels varied from 34.4 to 21.4. Conversely, the binding capacity of the three antibodies was also reduced in permeabilised cells after treatment with MDL28170, with significantly different values detected for anti-Dm-calpain and anti-CDPIIb antibodies (Supplementary data, Table I).

After investigating changes in levels of CALPs expression by flow cytometry, we aimed to detect calpain-like molecules in *P. serpens* by Western blotting using the same set of anti-calpain antibodies employed above. Anti-Dm-calpain antibody reacted with three polypeptide bands migrating at approximately 80 kDa, 70 kDa and 50 kDa (Supplementary data, Fig. 3). When anti-CAP5.5 and anti-CDPIIb antibodies were used, a single 50-kDa polypeptide was detected in both strains (Supplementary data, Fig. 3). The fact that *P. serpens* possesses molecules that share antigens with calpain-related enzymes is suggestive of these CALPs as being one of the targets of MDL28170. After MDL28170 treatment, the same polypeptide bands were detected for each antibody tested. However, the three bands detected with anti-Dm-calpain antibody and the 50-kDa band cross-reactive to anti-CAP5.5 antibody had their expression significantly reduced in MDL28170-treated cells (Supplementary data, Fig. 3). With this technique, we have already shown that MDL28170-treated *T. cruzi* epimastigotes had a decreased expression of CALPs cross-reactive to anti-Dm-calpain antibody (Branquinha et al. 2013), which may lead to the conclusion that the reduction in the expression of CALPs in trypanosomatids may prevail after treatment with the calpain inhibitor.

**Fig. 3:**
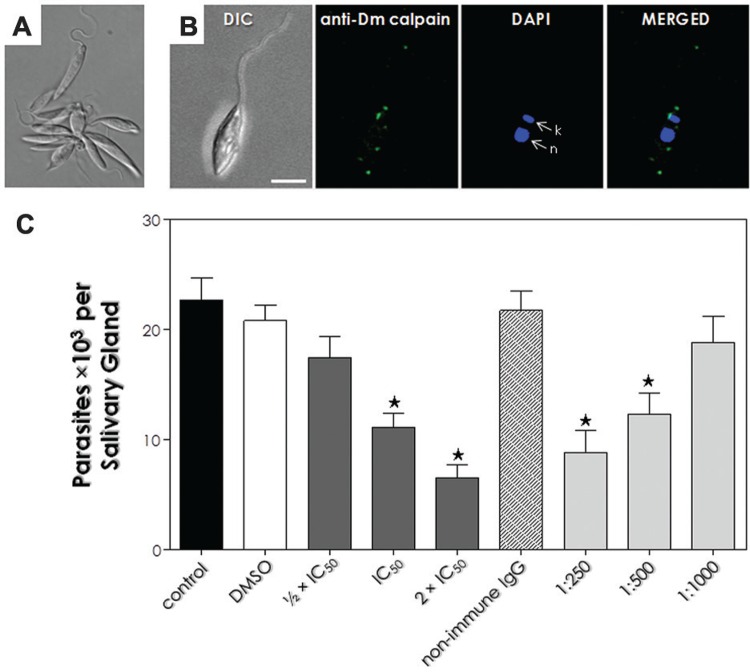
: effect of MDL28170 and anti-Dm-calpain antibody in the interaction process of *Phytomonas serpens* with the explanted salivary glands of *Oncopeltus fasciatus*. (A) Agglutination of parasites with anti-Dm-calpain antibody, which was only achieved up to 1:125 dilution. (B) Differential interference contrast microscopy (DIC) and confocal scanning images showing the double labeling of *P. serpens* cells for calpain-like proteins (CALPs) cross-reactive to anti-Dm-calpain antibody (green labeling) and DAPI (blue labeling). The labeling in non-permeabilised cells suggests the location of CALPs in the membrane and in the flagellum, and DAPI stained the nucleus (n) and the kinetoplast (k). At right, the overlay of anti-Dm-calpain and DAPI labeling. Bars: 5 µM. (C) Parasites (107 cells) were either pre-treated or not (control) with DMSO (diluent of the drug) or with the ½ x IC50, IC50 or 2 x IC50 values of MDL28170 for 4 h at 28ºC. In parallel, cells were pre-incubated with the anti-Dm-calpain (at 1:250, 1:500 and 1:1000 dilutions) or with a non-immune IgG antibody for 1 h. The parasite viability of the parasites was not affected by the treatments used in this set of experiments. Following interaction for 1 h, the salivary glands were washed and homogenised, and the released trypanosomatids were counted in a Neubauer chamber. The results are shown as the mean ± standard deviation of three independent experiments and the asterisks denote significant differences compared to control (p < 0.05).

The presence of a 50-kDa CALP that cross-reacted with anti-CAP5.5 antibody (Supplementary data, Fig. 3) is an interesting topic, since CAP5.5 protein was the first characterised member of calpain-related genes in a trypanosomatid, specifically in *T. brucei* (Hertz-Fowler et al. 2001). CAP5.5 is characterised by the similarity to the catalytic region of calpain-type peptidases and it has been shown to be both myristoylated and palmitoylated, suggesting a stable interaction with the cell membrane through interactions with the underlying microtubule cytoskeleton as well. The presence of CALPs that cross-react with anti-CAP5.5 antibody was already observed in *T. cruzi* epimastigote forms by flow cytometry on the cell surface but mainly in the intracellular milieu (Branquinha et al. 2013), in a similar pattern of distribution detected in the present work.

Another aspect that deserves consideration in the results presented herein is that a polypeptide band migrating at 80 kDa was also detected in *P. serpens* by cross-reactivity with the anti-Dm-calpain antibody (Supplementary data, Fig. 3), which was previously observed in different trypanosomatids (Branquinha et al. 2013). We postulate that the detection of the same protein band in trypanosomatids from different genera may suggest that these parasites share the same antigen with invertebrate calpain-related enzymes (Branquinha et al. 2013). Posteriorly, immunoprecipitation of this protein from the parasite lysate by anti Dm-calpain antibody followed by mass spectrometry analysis could be employed to definitely identify this 80-kDa protein.

Ersfeld et al. (2005) employed whole genome analysis and showed the presence of a large and diverse family of CALPs in the trypanosomatids *T. brucei* (14 members), *T. cruzi* (15 members) and *Leishmania* spp. (17 members). A search on the updated databases reveals that the actual number of CALPs is almost three times greater. However, since many of the CALPs present in trypanosomatids have amino acid substitutions in the well-conserved active site triad residues, it strongly suggests functions unrelated to proteolysis (Ersfeld et al. 2005, Branquinha et al. 2013).

*Protein sequence analysis* - Since we cannot exclude the possibility that the three anti-calpain antibodies used here may cross-react with proteins unrelated to CALPs in *P. serpens*, and once there is no *P. serpens* genome available, we performed a search in *Phytomonas* sp. isolate EM1 database of predicted proteins for homologues of the calpains from *D. melanogaster*, *T. brucei* CAP5.5 and lobster CDPIIb. There are evidences that the genus form a monophyletic group, being the EM1 isolate a sister group of *P. serpens* (Maslov et al. 2001). Hits corresponding to homologues with e-value ranging from 1e-83 to 1e-04, all corresponded to calcium-dependent cysteine peptidases homologous, had their theoretical molecular mass determined (data not shown). Among these hits, we selected those that presented a molecular mass compatible with the cross-reactivity detected in the Western blotting analysis. It is worth mentioning that typical calcium-binding domains from calpains were not found in these hits, as previously demonstrated by Ersfeld et al. (2005) that these domains are absent in trypanosomatids.

For *D. melanogaster* calpain, two homologues presented molecular masses around 80-70 kDa. For *T. brucei* calpain, only one homologue presented a molecular mass around 50 kDa, while for lobster calpain, no homologue was found with the molecular mass identified by Western blotting analysis (Supplementary data, Table II). At least one of the calpain domains cd00044 and pfam09149 was presented in the homologous sequences (Supplementary data, Table II). In this sense, cd00044 (or CysPc domain) corresponds to the domains IIa and IIb of the catalytic site of calpains, whereas pfam09149 (DUF1935 superfamily) is found in hypothetical proteins and calpains, with unknown function (Goll et al. 2003). The presence of at least one calpain-conserved domain as well as the theoretical molecular mass were consistent with the recognition by each antibody (Supplementary data, Table II).

When we performed a search for proteins, from *Phytomonas* sp. isolate EM1, containing homologues of CysPc domain from the *T. brucei* sequence, only six proteins were identified (sequence coverage range from 48 to 99%, and e-value from 5e-05 to 2e-126) using the Blastp algorithm at NCBI. This diminished number of calpains homologues could be related to the absence of identification of calpains that do not present this domain. Interestingly, the other three proteins categorised by gene ontology with “cysteine-type endopeptidase activity” after Blast analysis in UniProtKB, returned one protein (CCW64732.1) that shares similarities with cruzipain from *T. cruzi* (AAA30181.1), with an e-value of 20e-93 and 49% identity. Taken together, these data confirm the results shown in the present paper and previously published by our group (Santos et al. 2006, 2007) concerning the expression of cruzipain-like molecules in *P. serpens.*

Some of the proteins identified by Western blotting analysis were not found as calpain homologues in the predicted proteins from *Phytomonas* sp. isolate EM1, based on sequence similarities and molecular masses. The lack of identification could be related with post-translational modifications combined, or not, with the use of a set of predicted proteins obtained from a streamlined genome, not annotated, but the only one available at the moment of the analysis (Porcel et al. 2014).

Although we cannot state that the proteolytic activity identified in this paper is a true calpain family member at the present moment, we provided the first insights into the presence of a calcium-dependent cysteine peptidase activity in *P. serpens* promastigotes soluble extracts. Our group has previously demonstrated the purification and biochemical characterisation of a calcium-dependent cysteine peptidase from the culture supernatant of the insect trypanosomatid *A. deanei* (Branquinha et al. 2013). Among the many features in common with calpains, it highlights the absolute requirement of calcium for proteolytic activity and the cross-reaction with anti-Dm-calpain antibody. It is interesting to note that no proteolytic activity consistent with calpains has been described in *T. cruzi* or *L. amazonensis* (Branquinha et al., unpublished observations), which raises the question as to whether the retaining of its catalytic properties is a feature shared among non-mammalian parasites.

*Effects of MDL28170 and anti-Dm-calpain antibody on the interaction of P. serpens with O. fasciatus salivary glands* - We decided to test the influence of the calpain inhibitor and anti-Dm-calpain antibody on the interaction of *P. serpens* with explanted *O. fasciatus* salivary glands ex vivo. At first, we showed that live *P. serpens* promastigotes were agglutinated only when incubated with anti-Dm-calpain antibody up to 1:125 antibody dilution, suggesting that CALPs cross-reactive to this antibody are present at the parasite’s surface (Fig. 3A). Confocal immunofluorescence images corroborated the surface location of CALPs cross-reactive to anti-Dm-calpain antibody and also in the flagellum (Fig. 3B) in non-permeabilised cells, as previously detected in the flow cytometry analysis (Supplementary data, Table I). Pre-treatment of parasites with anti-Dm-calpain antibody at 1:250 and 1:500 dilutions also considerably impaired the parasite-salivary gland binding (64.5% and 43.3%, respectively), in comparison to control (Fig. 3C). When cells were either pre-treated with the anti-Dm-calpain antibody at 1:1000 dilution or with an irrelevant (non-immune) IgG (at 1:250 dilution), the interaction process was very similar to that obtained with non-treated parasites, confirming the anti-Dm-calpain antibody specificity (Fig. 3C).

The blockage of surface CALPs recognised by this antibody leading to a significant reduction on the capacity of adhesion to the salivary glands supports the notion that surface-located CALPs are important to this step of the life cycle. In a previous work from our group, we have also demonstrated the role of CALPs located at *T. cruzi* epimastigotes cell surface on the capacity to adhere to *R. prolixus* insect midgut by the pre-incubation with anti-Dm-calpain antibody, which reinforces the role of these molecules in the interaction of trypanosomatids with the invertebrate host (Branquinha et al. 2013). The mechanisms and factors underlying this phenomenon may reside in the less explored interactions governing parasite-host interplay.

We also aimed to assess the influence of the pre-treatment of parasite cells with MDL28170 in the interaction process. Parasites pre-treated with the IC_50_ and 2 x IC_50_ values of MDL28170 for 4 h showed a significant reduction (51.4% and 70%, respectively) in their capacity to adhere to the salivary glands (Fig. 3C). No significant change was observed when cells were pre-treated with the ½ x IC_50_ value of MDL28170. When cells were pre-incubated with DMSO (the solvent of the drug) in the volume corresponding to the highest concentration of the inhibitor, the interaction process was very similar to that obtained with non-treated parasites (Fig. 3C). In addition, the pre-treatment of parasites with MDL28170 or anti-Dm-calpain did not alter the parasite viability under the employed conditions used in this set of experiments (data not shown).

The significant reduction detected in the ability of promastigotes pre-treated with the IC_50_ and 2 x IC_50_ values of MDL28170 for 4 h to interact with *O. fasciatus* salivary gland can be correlated to the lower amount of anti-Dm-calpain cross-reactive CALPs. These results are consistent with the reported effect of the pre-treatment of *T. cruzi* epimastigote forms with MDL28170, which led to a reduction in CALPs expression and a significant reduction in the parasite binding to *Rhodnius prolixus* insect midgut (Branquinha et al. 2013).

**Fig. 4:**
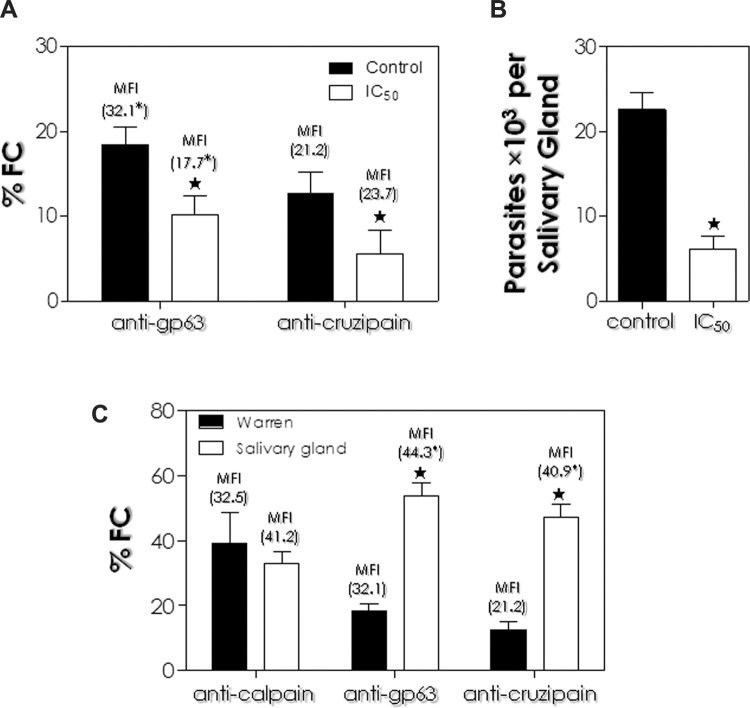
: correlation between the expression of surface gp63-like and cruzipain-like molecules and the interaction of *Phytomonas serpens* with salivary glands of *Oncopeltus fasciatus*. (A) Promastigotes were pre-treated or not (control) with the IC50 value of the calpain inhibitor MDL28170 for 24 h; then, paraformaldehyde-fixed cells were incubated in the presence of anti-gp63 and anti-cruzipain antibodies and analysed by flow cytometry. (B) Parasites were pre-treated or not (control) with the IC50 value of MDL28170 for 24 h. Following interaction for 1 h, the salivary glands were washed and homogenised, and the released trypanosomatids were counted in a Neubauer chamber. The results are shown as the mean ± standard deviation of three independent experiments. (C) Flow cytometry analysis showing calpain-like proteins (CALPs) cross-reactive to anti-Dm-calpain antibody, the gp63-like and cruzipain-like proteins expressed in *P. serpens* cultured in Warren medium and in parasites recovered from the interaction process with the salivary glands. In (A) and (C), data are expressed as the percentage of fluorescent cells (% FC) and also the mean of fluorescence intensity (MFI) levels. Representative data of the analysis of 10,000 cells from three experiments are shown. The stars and the asterisks highlight significant different values for % FC and MFI levels, respectively, compared to control in each system (p < 0.05). In (B), the results are shown as the mean ± standard deviation of three independent experiments and the star denotes significant difference compared to control (p < 0.05).

*Effects of MDL28170 on the expression of surface gp63-like and cruzipain-like molecules and on the interaction of P. serpens with O. fasciatus salivary glands* - In this set of experiments, the expression of gp63-like and cruzipain-like molecules at *P. serpens* cell surface was evaluated by flow cytometry in untreated and MDL28170-treated promastigotes, since both molecules were already described as potential adhesins expressed by the parasite to bind to salivary gland (Santos et al. 2007). This analysis revealed that both proteins had their expression significantly altered when parasites were subjected to the treatment with the calpain inhibitor at the IC_50_ value for 24 h (Fig. 4A). The percentage of cells labeled by anti-gp63 antibody was found to be decreased in the cell surface by 44% after MDL28170 treatment, while labeling of anti-cruzipain antibodies was reduced by 58%. In addition, a significant reduction in the MFI level of surface gp63-like molecules was detected after treatment with the calpain inhibitor, but no significant change in the MFI level of cruzipain-like molecules was found in the surface of MDL28170-treated parasites (Fig. 4A).

The influence of MDL28170 on the interaction of *P. serpens* with *O. fasciatus* salivary glands was previously tested when the calpain inhibitor was used at the IC_50_ value for 4 h (Fig. 3). Since a reduction in cell surface labeling with anti-gp63 and anti-cruzipain antibodies was detected after the 24 h treatment with the calpain inhibitor, this time interval was then used in the pre-treatment of cells with MDL28170 for the analysis of the interaction of *P. serpens* with *O. fasciatus* salivary glands. When compared to the 51.4% reduction in the capacity to adhere to salivary glands after treatment with MDL28170 for 4 h, as previously shown (Fig. 3), the 24 h treatment showed a 73.3% reduction in the capacity to adhere to *O. fasciatus* salivary glands (Fig. 4B). The significant reduction in gp63-like and cruzipain-like molecules detected in the *P. serpens* cell surface after a 24 h treatment with the IC_50_ value of MDl28170 with the concomitant greater reduction in the parasite adhesion to the salivary glands reinforces the role of these molecules in parasite adhesion. It is possible to postulate that MDL28170 treatment must affect biological events that depend on the surface exposition of certain proteins. In this context, our group has already demonstrated that gp63-like and cruzipain-like molecules found in *P. serpens* participate in several biological processes including adhesion to the salivary glands of *O. fasciatus*, a phytophagous insect experimental model (Santos et al. 2007).

Finally, promastigotes recovered from the interaction process with the salivary glands were processed for flow cytometry analysis in order to compare the expression of surface gp63-like and cruzipain-like molecules with parasites grown in Warren medium. This experiment revealed a significant increase in MFI levels both for surface gp63-like molecules (approximately 200%) and surface cruzipain-like molecules (approximately 250%) in parasites released from the salivary glands, which was paralleled by the significant increase in the labeling of these cells with anti-gp63 and anti-cruzipain antibodies. These data suggest an increased expression of surface gp63-like and cruzipain-like molecules in comparison to promastigote cells grown in Warren medium (Fig. 4C). However, the expression of CALPs cross-reactive to anti-Dm-calpain antibody was not significantly affected (Fig. 4C). The data presented in this study confirmed previous results from our group that correlated the interaction of trypanosomatids with the invertebrate hosts and the increased expression of molecules with roles in the interaction process. The expression of surface gp63-like molecules in the insect trypanosomatid *Herpetomonas samuelpessoai* was drastically enhanced after passage in the insect host model *Aedes aegypti* gut (Branquinha et al. 2013). In addition, *T. cruzi* cells isolated after passage in the insect vector *R. prolixus* presented a drastic increase in the expression of surface cruzipain (Uehara et al. 2012). Although the interaction step was not performed in vivo, our results revealed that a significant increase in the surface gp63-like and cruzipain-like expression was detected, which is very suggestive of their involvement in the interaction process of the parasite with the invertebrate vector. A possible explanation for this result is the selection of cells that naturally express higher levels of surface gp63-like and cruzipain-like proteins and that consequently display a better interaction with salivary glands of *O. fasciatus*. Although the relative levels of CALPs cross-reactive to anti-Dm-calpain antibody were not altered in *P. serpens* cells recovered from the interaction with salivary glands, it is possible that these molecules participate cooperatively in this process.

In conclusion, our results clearly showed that MDL28170 treatment of *P. serpens* promastigotes caused a pleiotropic response and revealed important functions in regulating parasite ultrastructure, the differential expression of proteins such as gp63-like, cruzipain-like, CALPs and calcium-dependent cysteine peptidases as well as the interaction of the parasite with the invertebrate host. However, the possible mechanisms underlying the effects of calpain inhibitors in trypanosomatids as well as the expression of distinct CALPs are still poorly understood. The findings of the present study underscore the potential of calpain inhibitors in the control of trypanosomatids infections and suggest studying their action on the parasites’ physiology in order to validate their potential contribution against these infections.
