# Supplementary material for: Susceptibility of Phytomonas serpens to calpain inhibitors in vitro: interference on the proliferation, ultrastructure, cysteine peptidase expression and interaction with the invertebrate host
Source: Mem Inst Oswaldo Cruz. 2016 Dec 1;112(1):31–43. doi: 10.1590/0074-02760160270 (PMC5224352; doi:10.1590/0074-02760160270)
Supplement: Supplementary file 1 [file 0074-0276-mioc-0074-02760160270-suppl01.pdf]

Mem Inst Oswaldo Cruz, Rio de Janeiro, Vol. *112*(1): 1-3, January 2016

TABLE I

Detection of cross-reactivity between calpain-like proteins from *Phytomonas serpens* and anti-calpain antibodies by flow cytometric analysis

Anti-calpain antibody	Non-permeabilised parasites				Triton X-100-permeabilised parasites			
	Control		MDL28170-treated cells (IC_{50} value)		Control		MDL28170-treated cells (IC_{50} value)	
	% FC	MFI	% FC	MFI	% FC	MFI	% FC	MFI
Anti-Dm-calpain	39.1 ± 9.7	37.1 ± 3.4**	37.8 ± 5,2	29.8 ± 7.2	85.4 ± 11.7*	191.2 ± 29.0**	69.0 ± 5,7	110.9 ± 21.7
Anti-CAP5.5	1.2 ± 0.9	15.2 ± 2.5	0.35 ± 0.1	11.7 ± 1.8	72.7 ± 7.6	26.4 ± 4.9**	64.5 ± 8.5	17.8 ± 1.4
Anti-CDPIIb	1.2 ± 0.9	15.5 ± 3.2	0.25 ± 0.2	12.9 ± 2.9	78.9 ± 10.4*	34.4 ± 6.7**	63.4 ± 8.6	21.4 ± 4.9

% FC: percentage of fluorescent cells; MFI: mean of fluorescence intensity; symbols (*, **) denote significant differences concerning either the % FC (*) or MFI (**) between control cells and cells treated with the IC_{50} value of MDL28170 ($p < 0.05$). Additionally, both the % FC and the MFI values for all tested antibodies were significantly different between non-permeabilised and Triton X-100-permeabilised parasite cells. Representative data of the analysis of 10,000 cells from three experiments are shown.

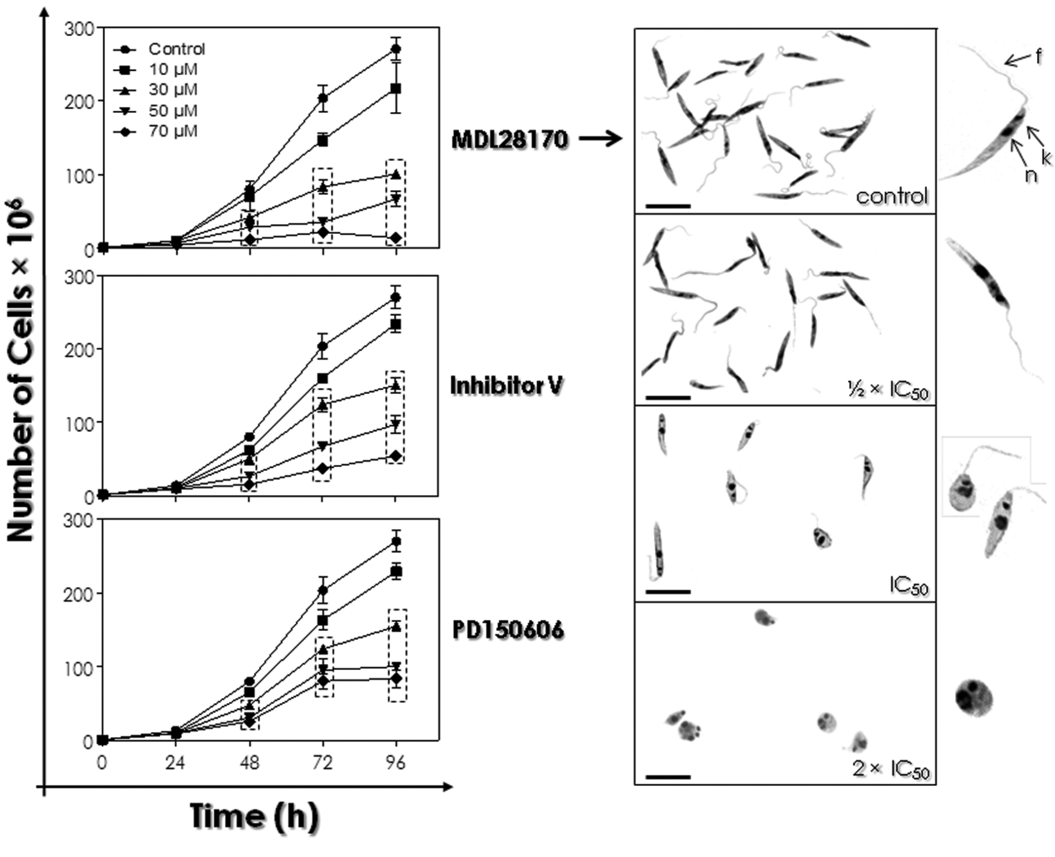

Fig. 1: effect of calpain inhibitors on the growth rate and the morphology of *Phytomonas serpens*. At left, the growth pattern of the parasite was followed in the absence (control) or in the presence of the calpain inhibitors MDL28170, inhibitor V and PD150606 at concentrations ranging from 10 to 70 µM. Each inhibitor was added to the cultures at 0 h, and the cells were counted daily. Data shown are the mean of three independent experiments performed in triplicate. The dashed boxes highlight growth rates significantly different from control ($p < 0.05$). The IC_{50} value of MDL28170 determined after 48 h of cultivation was 30.9 µM. At right, Giemsa-stained smears of *P. serpens* promastigote cells incubated in the absence (control) or in the presence of ½ x IC_{50}, IC_{50} or 2 x IC_{50} values of MDL28170 for 48 h. Promastigote forms present a kinetoplast (k) in the anterior end of the parasite, a central nucleus (n), an elongated cell body and a flagellum (f) attached to the parasite cell body. Note the reduction in the cell size, the round shape of the parasites and swollen of the cellular body as well as shortening and loss of the flagellum after treatment with MDL28170 at the IC_{50} and 2 × IC_{50} values. These morphological alterations were not observed after treatment of parasite cells with the ½ x IC_{50} value of the calpain inhibitor. The main parasite morphology(ies) observed in each system is(are) correspondingly shown outside of the boxes at the right. Bars: 10 µM.

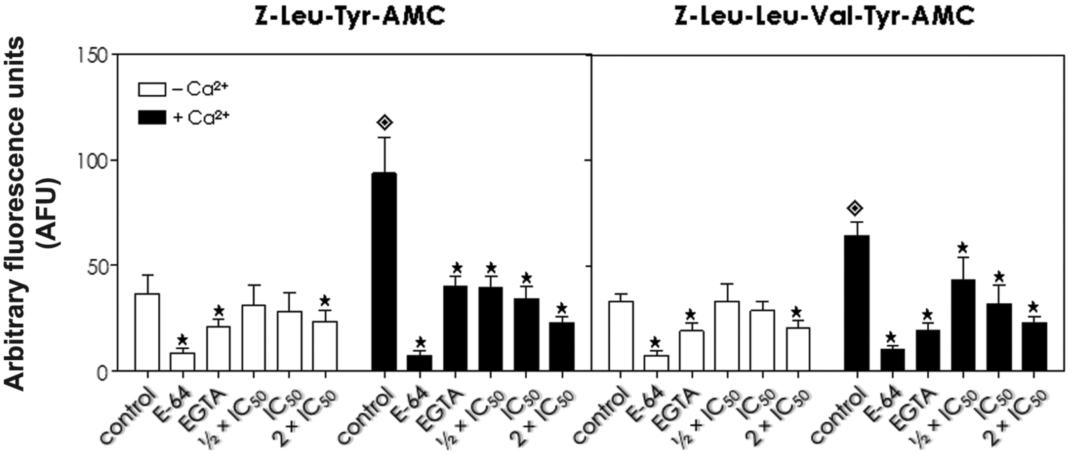

Fig. 2: effects of MDL28170 and calcium on *Phytomonas serpens* cysteine peptidase activity. *P. serpens* promastigotes were cultured in the absence (control) or the presence of the ½ x IC_{50}, IC_{50} or 2 x IC_{50} values of MDL28170 for 4 h. Then, the whole cellular extract (10 µg protein) was incubated with the fluorogenic substrates Z-Leu-Tyr-AMC and Z-Leu-Leu-Val-Tyr-AMC for 60 min at 37ºC in pH 7.0 in the absence ($-Ca^{2+}$) or in the presence ($+Ca^{2+}$) of 100 mM calcium chloride. Alternatively, the hydrolytic activity was measured in control cells in the presence of 10 µM E-64 or 1 mM EGTA. The results were expressed as arbitrary fluorescence units (AFU). The values represent the mean ± standard deviation of three independent experiments performed in triplicate. The diamonds highlight the significant differences between control systems in the absence or in the presence of calcium, and the asterisks denote systems treated with E-64, EGTA or MDL28170 that had a rate of substrate hydrolysis significantly different from the corresponding control ($p < 0.05$).

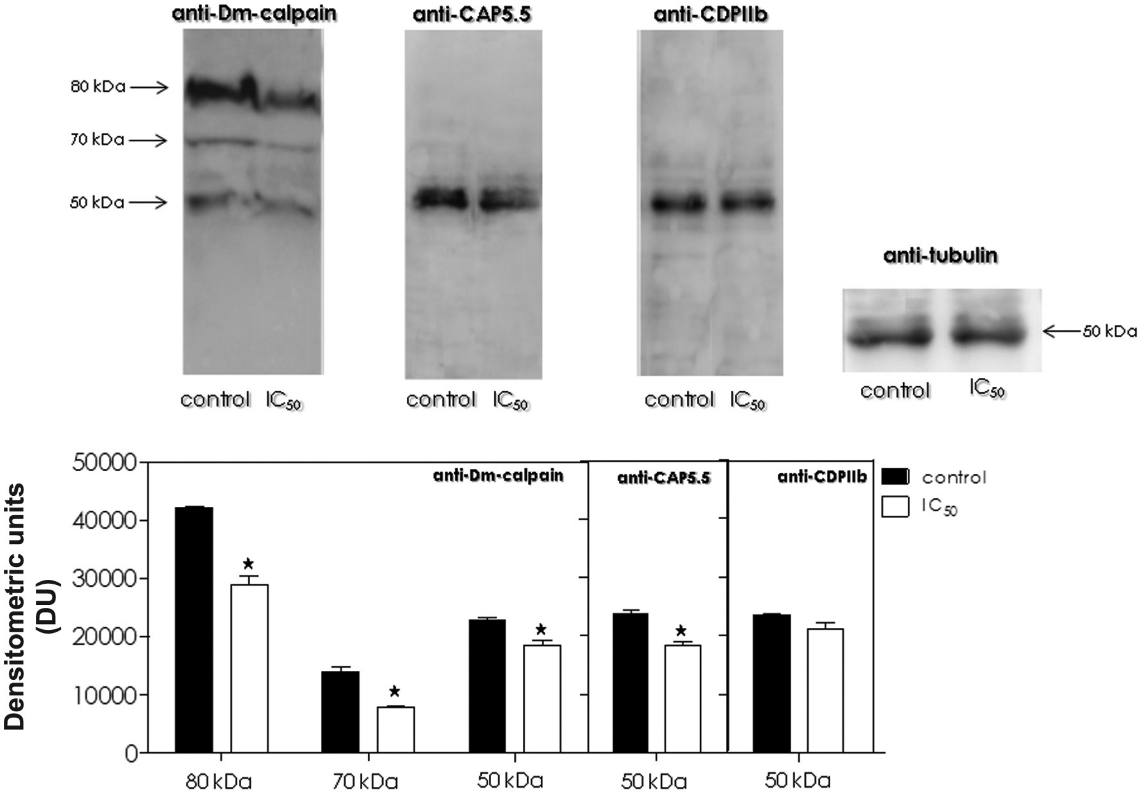

Fig. 3: detection of cross-reactivity between calpain-like proteins (CALPs) from *Phytomonas serpens* and anti-calpain antibodies. Upper panel, Western blotting showing the CALPs recognised in the whole cellular extract by the anti-Dm-calpain, anti-CAP5.5 and anti-CDPIIb antibodies. Promastigotes were pre-treated or not (control) with the IC_{50} value of the calpain inhibitor MDL28170 for 24 h. Anti-tubulin monoclonal antibody was used as a control for sample loading in the blots, revealing a protein band of 50 kDa in similar amount in control cells and after treatment with MDL28170. The apparent molecular masses, expressed in kilodaltons, of each detected band are shown. Lower panel, the densitometric analysis of the reactive bands detected in Western blotting is expressed as densitometric units (DU). The results represent means standard deviation of three independent experiments, and the asterisks denote statistic difference between control cells and promastigotes pre-treated with the IC_{50} value of MDL28170 ($p < 0.05$).

TABLE II

Possible *Phytomonas* sp. calpain-like proteins recognised by the antibodies employed in the present work and identified by BlastP analyses at NCBI

Anti-calpain antibody	Query protein acession #	Homologues in *Phytomonas* sp.					
		Acession #	Query cover (%)	Identity (%)	E value	Theoretical molecular mass (kDa)	Conserved domains
Anti-Dm-calpain	AHN56408.1	CCW65413.1	58	25	1e-27	88.5	cd00044 pfam09149
		CCW64468.1	37	26	2e-24	77.8	cd00044 pfam09149
Anti-CAP5.5	AAG48626.1	CCW60478.1	9	37	8e-06	55.0	pfam09149
Anti-CDPIIb	AAM88579.1	NI	NI	NI	NI	NI	NI

NI: not identified.
